# Antioxidant Activity during Tumor Progression: A Necessity for the Survival of Cancer Cells?

**DOI:** 10.3390/cancers8100092

**Published:** 2016-10-13

**Authors:** Mark A. Hawk, Chelsea McCallister, Zachary T. Schafer

**Affiliations:** Department of Biological Sciences, University of Notre Dame, Notre Dame, IN 46556, USA; Mark.A.Hawk.3@nd.edu (M.A.H.); Chelsea.M.McCallister.2@nd.edu (C.M.)

**Keywords:** antioxidant, reactive oxygen species, extracellular matrix, NADPH, Nrf2, pentose phosphate pathway, metastasis

## Abstract

Antioxidant defenses encompass a variety of distinct compounds and enzymes that are linked together through their capacity to neutralize and scavenge reactive oxygen species (ROS). While the relationship between ROS and tumorigenesis is clearly complex and context dependent, a number of recent studies have suggested that neutralizing ROS can facilitate tumor progression and metastasis in multiple cancer types through distinct mechanisms. These studies therefore infer that antioxidant activity may be necessary to support the viability and/or the invasive capacity of cancer cells during tumor progression and metastasis. Here, we discuss some of the accumulating evidence suggesting a role for antioxidant activity in facilitating tumor progression.

## 1. Introduction

Early evidence of a potential pro-tumorigenic role for antioxidant activity came from the results of a clinical trial in 1996 that surprisingly demonstrated worse outcomes for lung cancer patients who had a history of smoking and were given dietary supplements of β-carotene and vitamin A [1]. Following the publication of these unexpected data, additional studies were published that seemed to corroborate the idea that antioxidant activity could facilitate tumorigenesis [2,3,4]. However, a molecular mechanism that could clarify how cancer cells can benefit from antioxidant activity in a fashion that promotes tumor formation and/or progression remained elusive until the publication of several recent studies.

## 2. Antioxidant Activity in Extracellular Matrix-Detached Cells

One of the first clues regarding the aforementioned molecular mechanisms came from investigators studying luminal clearance in three-dimensional (3D) models of mammary morphogenesis. Using the MCF-10A model system, it is well established that cells populating the lumen lack contact with the extracellular matrix (ECM) and thus undergo anoikis (defined as ECM detachment-induced apoptosis) [5,6,7]. However, upon complete inhibition of anoikis (through overexpression of anti-apoptotic Bcl-2 proteins), the clearance of ECM-detached cells in the luminal space could still occur [8,9]. Thus, it stands to reason that there are non-apoptotic mechanisms involved in the death of ECM-detached cells in the luminal space. During the course of investigating how luminal, ECM-detached cells die in the absence of anoikis, investigators discovered that ROS levels are significantly elevated in these cells and are involved in caspase-independent cell death [10]. Interestingly, cell death in the luminal space could be inhibited through oncogenic signaling emanating from ErbB2, suggesting that cancer cells could improve their survival during ECM detachment by mitigating the deleterious effects of elevated ROS.

To more directly assess a possible link between oncogenic ErbB2 signaling and antioxidant activity, the investigators inhibited the NADPH-generating pentose phosphate pathway (PPP) and discovered that the ability of ErbB2 to promote survival was compromised [10]. Given that NADPH derived from the PPP is a significant source of antioxidant activity, these data imply that ErbB2 can promote the survival of ECM-detached cells through enhanced antioxidant activity. To further drive home this point, the investigators found that treating mammary acini with antioxidant compounds was sufficient to promote the survival of ECM-detached cells in the luminal space [10]. In aggregate, these data suggest that antioxidant activity (which can be acquired through signaling from the ErbB2 oncogene) promotes the survival of ECM-detached cells and thus, could be involved in facilitating tumor cell survival during metastasis.

While these findings represent one of the first studies to uncover the molecular mechanism by which antioxidant activity could facilitate tumorigenesis, there remained many interesting and important lines of investigation to pursue. Perhaps most significant: is it possible that cancer cells can utilize ubiquitously expressed ROS detoxifying enzymes to facilitate survival during periods of ECM detachment? Indeed, this is the case as overexpression of catalase or SOD2 is sufficient to promote luminal filling in 3D cultures of mammary acini [11]. Interestingly, the reduction of catalase expression by RNA interference did not impact the viability of ECM-attached cells but specifically compromised the survival of ECM-detached cells. Thus, there may be a therapeutic window in which to target catalase and specifically eliminate malignant ECM-detached cells. Consistent with this notion, previous studies have demonstrated that mice deficient in catalase are viable [12]. To extend upon these findings in vivo, the investigators explored whether a deficiency in catalase would compromise tumor formation in an experimental metastasis assay. Indeed, a reduction in catalase levels in MDA-MB-231 breast cancer cells did result in markedly less tumor formation in the lungs following a tail vein injection [11].

## 3. The Endogenous Antioxidant Program

Other studies examining the capacity of antioxidant enzymes to facilitate tumorigenesis have focused on Nrf2, a transcription factor that promotes the transcription of genes containing antioxidant response elements (AREs) such as catalase and SOD2 [13,14]. Perhaps most prominently, researchers recently discovered that the expression of K-Ras^G12D^, B-Raf^V619E^, and Myc^ERT2^ each elevated the Nrf2 antioxidant program by enhancing the transcription of Nrf2 and leading to a subsequent decrease in cellular ROS levels [15]. Expanding these findings further into lung and pancreatic cancer mouse models, the investigators used multiple genetic approaches to reduce Nrf2 expression. Blocking the expression of Nrf2 caused a significant reduction in tumor formation, disease burden and proliferation, and led to a substantive increase in median survival. Subsequent studies from this group suggest that the role of Nrf2 in pancreatic cancer involves Nrf2-mediated changes in EGFR signaling [16]. Thus, this study revealed that simultaneous inhibition of downstream EGFR effectors and the glutathione antioxidant pathway could mimic Nrf2 ablation and limit pancreatic cancer growth.

Researchers examining why diabetic patients are more susceptible to the development of various cancers also came to a similar conclusion on the Nrf2 pathway. Interestingly, it was discovered that drugs commonly utilized to treat patients with type 2 diabetes mellitus (e.g., dipeptidyl peptidase-4 (DPP-4i) inhibitors) can also function to promote sustained and enhanced activation of Nrf2 due to the inhibition of the Nrf2 antagonist KEAP1 [17]. While data from clinical trials suggest that treatment with DPP-4i compounds does not alter the risk for cancer development, DPP-4i treatment can promote metastasis in immunocompromised mice. An investigation of the molecular mechanism behind this enhanced metastasis revealed that Nrf2 is necessary for DPP-4i–mediated metastasis. Furthermore, Nrf2 activation was sufficient to promote metastasis in many of these same xenograft models. While additional studies need to be completed in other preclinical models (including in animals that are immunocompetent [18]), these data suggest that clinicians examining patients with (or at risk for development of) metastatic disease should conduct a comprehensive assessment of patient risk when prescribing anti-diabetes medications that can promote the activation of Nrf2 [19].

In addition to Nrf2, other studies examining the endogenous cellular antioxidant programs have focused on glutathione (GSH), the most abundant antioxidant present in human cells [20]. Using a mouse model of breast cancer, investigators demonstrated that genetic inhibition of glutamate cysteine ligase modifier (GCLM), a contributor to GSH synthesis, diminishes both tumor initiation and progression [21]. However, inhibition of GSH synthesis failed to inhibit tumor burden and progression if blocked beyond the time point of tumor onset, suggesting that antagonizing GSH solely is effective only prior to tumor formation. These data also raise the possibility that other antioxidant programs may compensate for a deficiency in a single antioxidant program. Indeed, this was the case in this particular study, as thioredoxin (TXN) levels were increased when GSH synthesis was blocked [21]. The utilization of small-molecule inhibitors to simultaneously block both TXN and GSH was an effective and synergistic strategy to reduce tumor growth in vivo. Thus, aspiring cancer cells may be eliminated by interfering with a single antioxidant program, but bona fide tumor cells can fall back on additional, redundant antioxidant programs to promote their survival.

In addition to the aforementioned antioxidant enzymes and detoxifying molecules discussed above, alterations in flux through distinct metabolic pathways can also dramatically alter ROS levels and thus contribute to tumorigenesis. One conspicuous example of this is the PPP, which (as discussed above) promotes antioxidant activity through NADPH production. Interestingly, this pathway has been linked to aerobic glycolysis, a term used to describe the propensity of cancer cells to rapidly metabolize glucose by glycolysis in oxygen-rich conditions [22]. It is now widely appreciated that a key regulator of aerobic glycolysis is pyruvate kinase M2 (PKM2) which was initially surprising due to its diminished enzymatic activity when compared to pyruvate kinase M1 (PKM1) [23]. However, a major clue into the function of PKM2 came from an elegant study demonstrating that pyruvate kinase inhibition is strikingly beneficial for cancer cells [24]. This inhibition results in the diversion of rapid glucose flux to the PPP where NADPH levels are increased, in turn driving down ROS levels. Thus, the observation by Otto Warburg (nearly 50 years ago) [25] that cancer cells utilized aerobic glycolysis may in fact be related (at least partially) to generating antioxidant activity.

## 4. Antioxidant Activity and Metastasis

As discussed above, antioxidant activity can promote the survival of ECM-detached cells and promote several other cellular behaviors associated with successful tumor metastasis. Thus, it may be the case that antioxidant activity may be a broad stimulant of cancer metastasis. Evidence from the aforementioned Nrf2 studies suggested this may be the case [15,17] and other investigations also seem to support this possibility. Researchers using a *Cre*-inducible oncogenic *Kras*^LSL^ model of lung cancer discovered that dietary supplementation of NAC (N-Acetyl Cysteine) or vitamin E (two antioxidants with distinct chemical structures that function to reduce ROS by entirely distinct mechanisms) augments tumor progression and compromises survival [26]. This enhanced tumor progression was found to be dependent on the reduction of p53 activity.

Intriguingly, dietary supplementation with NAC or vitamin E was found to promote metastasis in other types of cancer as well. Using a mouse model of malignant melanoma, antioxidant supplementation was found to increase lymph node metastases without any impact on primary tumor formation [27]. Mechanistically, melanoma metastasis in this model was driven by RhoA-mediated cellular invasion. Similar findings were observed in a distinct mouse model (NOD-SCID-*Il2rg*^−/−^ (NSG)) of melanoma metastasis where NAC was supplemented regularly via subcutaneous injection [28]. NAC injection of NSG mice did not impact primary tumor formation but did substantially influence successful metastasis. In this model, the link between antioxidant activity and metastasis involved significant metabolic adaptation that involved a reliance on the generation of NADPH from the folate pathway. Thus, even within the same type of cancer, antioxidant activity seems to have the capacity to trigger multiple mechanisms to facilitate the dissemination of metastatic cancer cells.

## 5. Conclusions

**Figure 1:**
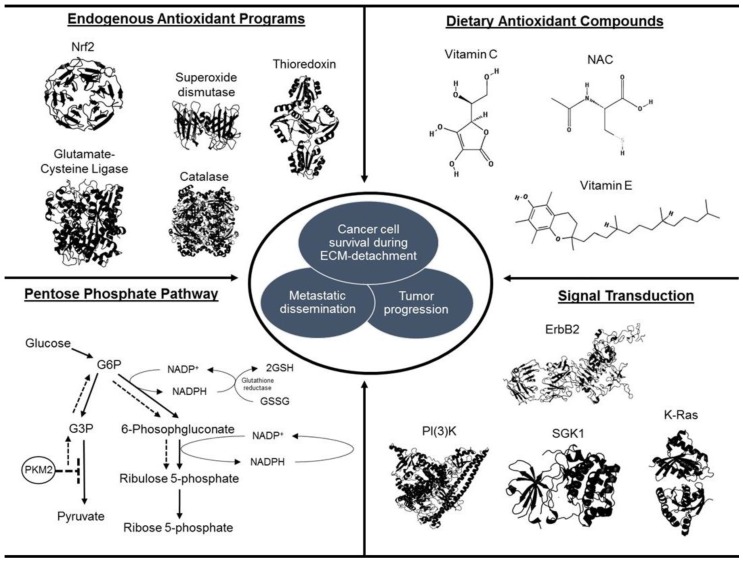
Mechanisms of antioxidant generation during cancer development. A wide variety of endogenous antioxidant pathways exist within cells which mitigate oxidative stress to enhance cell survival. The above pathways are paramount to augment survival in the absence of matrix attachment, tumor progression, and metastatic dissemination. All protein structures were retrieved from the RSCB Protein Data Bank [30] and compounds were retrieved from PubChem Compound.

In aggregate, although there have been incredible advances in the understanding of the role of antioxidants in tumor progression (summarized in Figure 1), the context-dependent nature of the studies addressed in this review highlight the need for additional research on antioxidants, tumor progression, and metastasis. A recent study on in colorectal cancer emphasizes the incredibly complex nature of understanding how antioxidant activity affects cancer cells. Unexpectedly, it was discovered that high doses of vitamin C (which has well-documented antioxidant activity) can lead to an increase in intracellular ROS in cells with activating mutations in K-Ras or B-Raf [29]. This increase in ROS is due to the fact that oxidized vitamin C (dehydroascorbate (DHA)) is preferentially taken up by cancer cells. The increase in cellular ROS of cancer cells that take up DHA is therefore a consequence of the enhanced antioxidant burden necessary to reduce DHA to vitamin C. As a result, the enhanced levels of ROS can lead to a bioenergetic crisis (due to inactivation of GADPH) and consequent cell death. Thus, even though it is clear that these cells rely on antioxidant activity for survival, they can counter-intuitively be killed by an agent with antioxidant properties that at high doses can function to enhance ROS. It is clear that antioxidant activity can have divergent effects on cancer cells depending on the cellular context. Nonetheless, the multitude of studies discussed here suggest that patients diagnosed with (or at risk for) cancers should avoid unnecessary supplementation of antioxidants in order ensure that enhanced antioxidant activity does not inadvertently facilitate tumor progression and metastasis.
